# Attitudes toward eating disorders among primary care physicians

**DOI:** 10.1080/02813432.2026.2755219

**Published:** 2026-11-02

**Authors:** Lena Wendel, Ata Ghaderi, David Iggman, Elisabeth Welch, Per Kristiansson

**Affiliations:** ^a^ Department of Public Health and Caring Sciences/Family Medicine and Preventive Medicine, Uppsala University, Uppsala, Sweden; ^b^ Center for Clinical Research Dalarna, Falun, Sweden; ^c^ Department of Clinical Neuroscience, Division of Psychology, Karolinska Institutet, Stockholm, Sweden; ^d^ Department of Public Health and Caring Sciences/Clinical Nutrition and Metabolism, Uppsala University, Uppsala, Sweden; ^e^ Department of Women’s and Children’s Health, Global Health and Migration Unit, Uppsala University, Uppsala, Sweden

**Keywords:** Eating disorders, attitudes, primary health care, primary care physicians, early detection

## Abstract

**Background:**

Eating disorders are severe conditions associated with high morbidity, mortality and socioeconomic burden. Primary care physicians are central in their early detection and treatment, but clinicians have been found to have limited knowledge and even negative attitudes toward eating disorder patients. Most studies have focused on anorexia nervosa (AN) and bulimia nervosa (BN), while less is known about attitudes toward binge eating disorder (BED) and avoidant/restrictive food intake disorder (ARFID).

**Aim:**

To examine primary care physicians’ attitudes toward eating disorders and assess whether education and clinical experience predict comfort in management and willingness to work with affected patients.

**Methods:**

A cross-sectional survey was conducted among primary care physicians (*n* = 191) attending a mandatory seminar. Attitudes toward AN, BN, BED and ARFID were assessed using Likert-type items based on prior research. Ordinal logistic regression analyses examined associations between education, experience and selected attitudes.

**Results:**

Overall attitudes were similar across diagnoses. Only 14–21% reported enjoying working with eating disorder patients. Education was consistently associated with greater comfort in management and enjoyment of working with these patients, whereas clinical experience was more strongly associated with comfort in management than with enjoyment. Familiarity with ARFID was low, reflected in reduced response rate (14.6%).

**Conclusions:**

Attitudes toward eating disorders were broadly similar across diagnoses, but positive attitudes toward working with affected patients were uncommon. Education was consistently associated with more favorable attitudes and greater confidence in management, highlighting the potential value of continuing professional education and support for eating disorder care in primary care.

## Introduction

Eating disorders are complex conditions with increasing prevalence, associated with significant mortality [1], morbidity [2] and socioeconomic burden [3]. According to DSM-5 [4], the major eating disorder diagnoses are anorexia nervosa (AN), bulimia nervosa (BN), binge eating disorder (BED) and avoidant/restrictive food intake disorder (ARFID), the latter two having been introduced as distinct diagnoses in DSM-5 [5]. Although AN is most common among adolescent and young adult females, eating disorders affect individuals of all ages and genders, and BED has a more balanced sex distribution [6,7]. Many individuals with eating disorders remain unidentified and consequently do not receive evidence-based treatment [8,9].

Previous research has demonstrated limited knowledge, low confidence in management, negative attitudes toward patients with eating disorders, and a need for additional training among healthcare providers [10–13]. Similarly, inadequate training, uncertainty regarding referral pathways, and limited knowledge and confidence have been identified as key barriers to the recognition and management of eating disorders among non-specialist healthcare professionals [14–16]. Beyond knowledge and training deficits, primary care physicians play a key role in the detection and management of eating disorders [17,18]. Many cases are identified during consultations for other health concerns; yet, challenges remain regarding screening, recognition and management pathways [19–21]. In Sweden, primary care often serves as the first point of contact and gateway to specialized eating disorder services. Primary care physicians are responsible for recognizing possible eating disorders, conducting an initial assessment, evaluating medical risk, initiating investigations and referring patients when specialist treatment is required.

Previous research has documented stigmatizing attitudes and negative perceptions of patients with eating disorders among healthcare professionals [10,22,23]. Two survey-based studies among general practitioners [24] and resident physicians [25] found limited knowledge and negative attitudes toward patients with AN and BN, with only about one in 10 respondents reporting comfort in managing or enjoyment in working with these patients. Limited knowledge has also been associated with greater insecurity in identifying and managing eating disorders and with more negative attitudes toward affected patients [16]. Although primary care physicians commonly encounter patients with eating disorders, relatively few studies have examined their attitudes toward eating disorders. Most studies have been conducted in the United Kingdom and the United States [24,25]. Differences in healthcare organization, access to specialist services, professional training, and the role of primary care may limit the generalizability of these findings to the healthcare setting in Sweden and similar countries, underscoring the need for country-specific research. Historically, educational efforts, clinical guidelines and research have predominantly focused on AN and BN, particularly in Sweden, where healthcare has traditionally relied on ICD-10 classification systems. Lower familiarity and limited training have been associated with lower confidence and less favorable attitudes toward eating disorders [15,25], suggesting that attitudes may differ between established and newer diagnostic categories. Recent evidence, including a systematic review and a national survey of primary care professionals, has identified barriers to the recognition and management of eating disorders in primary care and has expanded knowledge regarding attitudes toward BN and BED [16,20]. However, these studies focused primarily on BN and BED, while evidence regarding primary care physicians’ perceptions and management of ARFID remains limited.

Consequently, knowledge about attitudes toward the broader spectrum of DSM-5 eating disorders in primary care, particularly within a Swedish context, remains limited. The aim of this study was to examine primary care physicians’ attitudes toward AN, BN, BED and ARFID and to explore whether education and clinical experience were associated with physicians’ attitudes. Based on previous research, we hypothesized that physicians would report limited confidence in managing patients with eating disorders and relatively low enjoyment of working with this patient group. We further hypothesized that attitudes would be less favorable toward the newer diagnoses BED and ARFID than toward AN and BN, and that greater education and clinical experience would be associated with more positive attitudes.

## Methods

### Study design, setting and participants

This was a cross-sectional survey-based study using REDCap (Research Electronic Data Capture) [26] which is a web application for building and managing surveys to collect and analyze data. We conducted the study in late 2024 at the bi-annual mandatory seminar for physicians working in primary health care in Region Dalarna, Sweden, encompassing primary healthcare centers in both rural and urban areas. Region Dalarna was chosen because one of the investigators works within the regional primary care organization and was therefore able to access a seminar attended by virtually all physicians working in primary care. This provided a unique opportunity to employ a census-based approach targeting the entire accessible physician population in the region.

All employed doctors were given paid leave from clinical work to participate on one of two consecutive seminar days with identical content. We were given about 15 min each day to briefly inform about the study and for the participants to complete the web-based survey. All physicians attending the seminar, either on site or online, were invited to participate. The eligible population consisted of 191 physicians. Participants included general practitioners (specialists in family medicine), residents and interns. The majority attended on site, while a minority participated online. No distinction was made between participants who attended in person and those who participated online. The total number of physicians in this region is about 200.

### Instruments

The questionnaire consisted of two sections: (1) demographic and professional background questions and (2) attitude items relating to eating disorders (Appendix 1). The first part of the survey consisted of demographic questions, such as sex and age. Level of competence was operationalized as formal professional qualification within the Swedish medical training system (intern, resident or specialist physician), rather than as a measure of clinical competence. Additional variables included experience of complementary missions within primary care, defined as voluntary additional clinical roles or responsibilities alongside regular primary care practice, where encounters with patients with eating disorders may be more common (e.g. maternity care, child health services, school health services and youth health centers), eating disorder-related training and experience of assessing or treating patients with an eating disorder.

Attitude questions were based on previous studies [24,25], with three additional items addressing the role of primary care physicians in the care pathway (Appendix 1). The items assessed perceptions of illness severity, patient responsibility, treatment effectiveness, quality-of-life impact, the role of primary care, perceived support and confidence and enjoyment in managing patients with eating disorders. The same set of attitude items was presented separately for AN, BN, BED and ARFID, enabling respondents to evaluate each diagnosis independently. Thus, attitudes toward the four diagnostic categories were assessed using parallel items specific to each diagnosis. The attitude items were rated on a Likert-like scale from 1 (strongly agree) to 5 (strongly disagree). For ARFID, a gatekeeping question was included because this diagnosis was expected to be less familiar to primary care physicians than AN, BN and BED. Specialist clinics for treatment of eating disorders started to use the DSM-IV research criteria for BED, and categorized patients based on them as early as in 1999 [27], thanks to the National Swedish Registry for eating disorders. BED has been known in Sweden for much longer time than ARFID, although both became formal diagnoses in DSM-5 in 2013 [4]. Participants were asked whether they considered themselves sufficiently familiar with ARFID to answer the subsequent attitude questions. Before conducting the study, the questionnaire was pilot-tested on 14 primary care physicians working in three other regions in Sweden. Based on the feedback, some minor linguistic revisions were made.

### Study size

The study aimed to include the entire accessible population of physicians attending the regional primary care seminar. Consequently, no formal sample-size calculation was performed.

### Statistical methods

Descriptive statistics such as frequencies and means were used to present the majority of the results. In the results, the Likert scale was dichotomized into two alternatives: those who agreed (‘strongly agree’, ‘agree’) and those who did not agree (‘neither agree nor disagree’, ‘disagree’, ‘strongly disagree’). The neutral response category was grouped with the non-agreement categories because the aim was to distinguish explicit agreement from all other responses. This approach was chosen to facilitate interpretation and to provide a clearer and more accessible presentation of the results.

Ordinal logistic regression analyses using the original ordinal coding of the Likert scale were conducted to examine how education and experience were associated with the attitudes ‘Comfortable in management’ and ‘Enjoy working with these patients’.

A directed acyclic graph (DAG) using DAGitty [28] was constructed to identify a minimally sufficient set of confounders for adjustment in the logistic regression analyses. The DAGs were based on a priori assumptions regarding the relationships between physician characteristics, education, experience and attitudes toward eating disorders. Because separate DAGs were developed for different exposure–outcome relationships, the resulting covariate adjustment sets differed between models. All the analyses were conducted in SAS and Jamovi.

*Ethical considerations*. An application was submitted to The Swedish Ethical Review Authority (Dnr 2024-03823-01). The authority determined that the study did not require formal ethical review because no intervention was performed and no personal data covered by the Swedish Ethical Review Act were processed. A favorable advisory opinion was issued and no ethical objections to the project were raised. No identifying information was collected. All participants provided informed consent electronically before accessing the survey.

## Results

### Response rate

According to the participation list, 161 physicians attended on site and an additional 30 online. Of the total 191 participants, 178 (93.2%) completed the demographic questions and 174 (91.1%) completed the attitude section, which constituted the main analytical sample. For ARFID, the gatekeeping question reduced the respondents to 26 individuals (14.6%).

### Demographics

Table 1 shows the characteristics of the study population. The participants ranged in age from 25 to 75 years, with a mean age of 40.6 years. Regarding continuing education on eating disorders during the previous five years, the majority of the participants, 107 (60%) had none. As for experience, 118 (66%) reported having assessed, treated or referred 1–5 patients with an eating disorder in the last 5 years, compared to the 33 (18%) that stated they had not managed any ED patients in the last five years.

**Table 1.** Characteristics of the 178 participating physicians.

Characteristic	
Age (years)	40.6 (10.2)
Female sex	102 (57)
Level of competence	
Intern/resident	80 (45)
Specialist	98 (55)
Complementary mission within primary care^a^	
Maternity care	44 (25)
Children’s health care	84 (47)
School health service	3 (2)
Youth health center	15 (8)
None of the above	83 (47)
No. of complementary mission within primary care	
0	83 (47)
1	53 (30)
2	34 (19)
3	7 (4)
4	1 (1)
Continuing education in eating disorders (no. of hours)^b^	
0	107 (60)
1–5	61 (34)
6–10	5 (3)
>10	5 (3)
Experience of managing patients with an eating disorder (no.)^b^	
0	33 (18)
1–5	118 (66)
6–10	20 (11)
>10	7 (4)

Frequency (%) and mean (standard deviation) are presented.

^a^ During the past 5 years. Denominator is 178 participants. Each participant may hold more than one side mission.

^b^ During the past 5 years.

### Attitudes toward ED

Table 2 shows the stated attitudes for each of the different eating disorder diagnoses (AN, BN, BED, ARFID). Physicians expressed broadly similar attitudes across AN, BN, BED and ARFID for most questionnaire items. The most notable differences between diagnoses were observed for perceptions of the disorders as severe and enduring, treatment effectiveness, patients’ responsibility for their condition, and clarity of the physician’s role in the care pathway.

**Table 2.** The number and percentage (%) of physicians who agreed or strongly agreed with each statement.

Attitudes	AN	BN	BED	ARFID
Comfortable in management	53 (30.5)	42 (24.1)	31 (17.8)	9 (34.6)
Enjoy working with these patients	36 (20.7)	32 (18.4)	25 (14.4)	5 (19.2)
Disease being psychological	102 (58.6)	96 (55.2)	98 (56.3)	13 (50.0)
Symptoms usually disappear without treatment	25 (14.4)	25 (14.4)	22 (12.6)	6 (23.1)
Disease being severe and enduring	122 (70.1)	94 (54.0)	86 (49.4)	12 (46.15)
Patients being responsible for their condition	14 (8.1)	33 (19.0)	34 (19.5)	1 (3.9)
Disease effect on life quality of the patient	174 (100)	174 (99.4)	166 (95.4)	25 (96.2)
Disease effect on family	174 (100)	169 (97.1)	148 (85.1)	25 (96.2)
Effect of treatment	14 (8.1)	113 (64.9)	82 (47.1)	9 (34.6)
Patients can control their symptoms	59 (33.9)	65 (37.4)	72 (41.4)	1 (3.9)
I play an important role for these patients	106 (60.9)	86 (49.4)	67 (38.5)	7 (26.9)
I feel supported by colleagues	35 (20.1)	32 (18.4)	23 (13.2)	5 (19.2)
I consider my role in the care chain as clear	39 (22.4)	42 (24.1)	37 (21.3)	2 (7.7)

The total *n* was 174 for AN, BN and BED, while the statements regarding ARFID were responded to by only 26 participants.

Table 2 also shows the level of agreement across the diagnoses regarding the attitude items. High levels of agreement with the attitude item were seen concerning the attitudes ‘Disease effect on life quality of the patient’ and ‘Disease effect on family’. Low levels of agreement were seen regarding ‘Enjoying working with these patients’, ‘Symptoms usually disappear without treatment’, ‘Patients being responsible for their condition’ and ‘I feel supported by colleagues’. No distinctly high or low levels of agreement across the attitudes were observed regarding ‘Effect of treatment’ and ‘Patients can control their symptoms’.

### Association between demographics and attitudes

Tables 3a and 3b show the results of the logistic regression analyses, and the adjustments done according to the DAGs. Table 3a shows the results regarding the attitude ‘Comfortable in management’. The participants’ comfort in managing patients with AN was significantly predicted by education (OR = 2.17, CI = 1.40–3.38, *p* < 0.01) and experience (OR = 1.89, CI = 1.25–2.88, *p* = 0.003). In other words, physicians with longer education and more experience reported being more comfortable in managing patients with AN. For BN the participants’ comfort was predicted by education (OR = 1.58, CI = 1.06–2.40, *p* = 0.028), as it was for BED (OR = 1.83, CI = 1.20–2.83, *p* = 0.005). Concerning ARFID, experience was a significant predictor (OR = 4.09, CI = 1.40–14.13, *p* = 0.015).

**Table 3a.** Results ordinal logistic regression models for examining association and effect estimation of the exposure variable (education and experience) on the outcome variable ‘comfortable in management’ regarding anorexia nervosa (AN), bulimia nervosa (BN), binge eating disorder (BED) and avoidant/restrictive food intake disorder (ARFID).

	Comfortable in management					
	Simple logistic regression			Multiple logistic regression		
	OR	CI	*p*	OR	CI	*p*
AN						
Education	1.80	1.19–2.74	**0.005**	2.17^a^	1.40–3.38	**<0.01**
Experience	1.90	1.27–2.86	**0.002**	1.89^b^	1.25–2.88	**0.003**
BN						
Education	1.38	0.94–2.04	0.105	1.58^a^	1.06–2.40	**0.028**
Experience	1.49	1.02–2.19	**0.040**	1.41^b^	0.94–2.13	0.097
BED						
Education	1.69	1.13–2.55	**0.012**	1.83^a^	1.20–2.83	**0.005**
Experience	1.08	0.71–1.63	0.726	0.94^b^	0.61–1.44	0.778
ARFID						
Education	1.50	0.70–3.51	0.319	1.87^a^	0.81–4.87	0.161
Experience	3.16	1.20–9.49	**0.027**	4.09^b^	1.40–14.13	**0.015**

Odds ratios (ORs) and 95% confidence intervals (CIs) are presented (*n* = 174 participating physicians).

Bold values indicate statistically significant p-values (*p* < 0.05).

^a^ Adjusted for age.

^b^ Adjusted for age, sex, education and complementary missions.

The attitude ‘Enjoy working with these patients’ was significantly predicted by education (Table 3b), for all four eating disorder diagnoses; AN (OR = 1.67, CI = 1.09–2.57, *p* = 0.018), BN (OR = 1.85, CI = 1.21–2.84, *p* = 0.004), BED (OR = 1.92, CI = 1.26–2.98, *p* = 0.003) and ARFID (OR = 3.28, CI = 1.34–9.21, *p* = 0.013).

**Table 3b.** Results ordinal logistic regression models for examining association and effect estimation of the exposure variable (education and experience) on the outcome variable ‘enjoy patient group’ regarding anorexia nervosa (an), bulimia nervosa (BN), binge eating disorder (BED) and avoidant/restrictive food intake disorder (ARFID).

	Enjoy patient group					
	Simple logistic regression			Multiple logistic regression		
	OR	CI	*p*	OR	CI	*p*
AN						
Education	1.46	0.98–2.20	0.067	1.67^a^	1.09–2.57	**0.018**
Experience	1.43	0.95–2.15	0.09	1.28^b^	0.84–1.95	0.252
BN						
Education	1.57	1.05–2.36	**0.029**	1.85^a^	1.21–2.84	**0.004**
Experience	1.33	0.88–2.00	0.177	1.18^b^	0.77–1.80	0.458
BED						
Education	1.69	1.13–2.55	**0.012**	1.92^a^	1.26–2.98	**0.003**
Experience	1.08	0.71–1.63	0.726	0.92^b^	0.60–1.42	0.718
ARFID						
Education	1.91	0.87–4.69	0.123	3.28^a^	1.34–9.21	**0.013**
Experience	1.73	0.70–4.43	0.239	1.47^b^	0.56–4.03	0.442

Odds ratios (ORs) and 95% confidence intervals (CIs) are presented (*n* = 174 participating physicians).

Bold values indicate statistically significant p-values (*p* < 0.05).

^a^ Adjusted for age and sex.

^b^ Adjusted for sex, education and complementary missions.

## Discussion

### Main findings

The results provide insights into primary care physicians’ attitudes toward eating disorders. Attitudes were largely similar across diagnoses, although differences were observed regarding perceptions of the disorders, treatment, their role in the care chain, and patient responsibility. Most physicians agreed that eating disorders negatively affect the quality of life of both patients and their families. However, enjoyment of working with these patients was low across diagnoses, ranging from 14.4% for BED to 20.7% for AN. Education was associated with both greater enjoyment of working with patients with eating disorders and higher comfort in managing them, suggesting a relationship between education and more positive attitudes. Yet, only a minority had received continuing education on eating disorders.

### Comparison with literature

In contrast to previous studies focusing primarily on AN and BN, we examined attitudes toward the broader spectrum of DSM-5 eating disorders. Although attitudes were generally similar across diagnoses, some differences emerged. For example, physicians reported greater comfort in managing AN than BED, possibly reflecting lower familiarity with BED. This interpretation is consistent with previous research showing that healthcare providers often report limited knowledge and skills related to eating disorders and feel insufficiently prepared to identify and manage these conditions [11]. While comfort levels appeared relatively high for ARFID, the small number of respondents familiar with the diagnosis warrants cautious interpretation.

Differences were also observed regarding the statement ‘I play an important role for these patients’, where the majority agreed with respect to AN, but less than two-fifths regarding BED. This finding is noteworthy, particularly concerning BED, whose somatic complications related to the metabolic syndrome [29] are largely managed in primary care.

In contrast to Anderson et al. [25], only a minority of our participants perceived patients as responsible for their condition. This difference may partly reflect differences in study populations, as Anderson et al. surveyed residents from several specialties whereas our sample consisted of primary care physicians. In contrast, agreement with the statement ‘Enjoy working with these patients’ was similarly low in both studies, with only 14–21% of respondents expressing agreement. The complexity of eating disorders may contribute to negative reactions among healthcare professionals, a finding previously reported in studies [22,23]. Contrary to our hypothesis, attitudes toward BED and ARFID were not consistently more negative than those toward AN and BN. This suggests that physicians may perceive eating disorders as a relatively homogeneous group despite differences in symptom presentation and diagnostic history. It may also reflect increased awareness of BED and ARFID since their introduction in DSM-5. Nevertheless, healthcare professionals continue to report uncertainty regarding the recognition and management of BED, and knowledge gaps remain an important barrier in primary care [16].

An additional finding of interest was that while greater experience predicted higher comfort levels for several diagnoses, it was not a significant predictor of enjoyment of working with patients with BN, BED or ARFID, and showed only a non-significant trend for AN. This suggests that repeated clinical exposure may increase physicians’ confidence without necessarily changing their affective attitudes toward this patient group. Eating disorders are often characterized by chronicity, treatment ambivalence and complex clinical presentations, which may remain challenging even for experienced clinicians. In contrast, education was consistently associated with greater enjoyment of working with patients across all diagnoses. Taken together, these findings suggest that knowledge acquisition and professional understanding may be more closely related to positive attitudes than clinical exposure alone. This interpretation is consistent with previous studies reporting insufficient training in eating disorders and a need for further education to improve knowledge and confidence in clinical management [11].

The finding that many physicians reported uncertainty regarding their role in the care pathway may be clinically important, as primary care serves as the main entry point to specialist eating-disorder services. Unclear responsibilities and referral pathways may delay recognition and treatment. Similar barriers have previously been identified in primary care settings [19].

### Strengths and limitations

A major strength of this study was the high response rate, exceeding 90%, achieved by surveying primary care physicians attending a mandatory educational event. This enabled a near-census of physicians within the region and minimized selection bias. Another strength is the inclusion of attitudes toward the broader spectrum of DSM-5 eating disorders, including BED and ARFID, diagnoses that have received limited attention in previous primary care research.

Nevertheless, several limitations should be acknowledged. The study was conducted in a single Swedish healthcare region, which may limit generalizability to other settings.

One limitation of this study is the lack of robust psychometric evaluation of the questionnaire. The instrument was based on previously published studies and designed to capture several distinct attitude domains rather than a single underlying construct. Consequently, internal consistency measures such as Cronbach’s alpha were not calculated, as some domains contained too few items for meaningful analysis.

Another limitation is that the survey relied on structured questionnaire items and did not include open-ended questions. Consequently, while the study identified patterns in physicians’ attitudes, it could not explore the experiences or contextual factors underlying them.

Although the survey was piloted and revised based on participant feedback, some items remained open to interpretation. For example, the item assessing physicians’ outlook on treatment may have reflected knowledge of treatment outcomes as well as attitudes. Another limitation concerns ARFID, for which only 14.6% of participants (*n* = 26) completed the attitude items following the gatekeeping question. While this was an intentional design choice and indicates limited familiarity with ARFID among participants, the low response rate reduces confidence in these findings. In contrast, response rates for AN, BN and BED were high, strengthening confidence in the results for these diagnoses.

Furthermore, a limitation of the study is the dichotomization of Likert-scale responses, which inevitably resulted in a loss of informational richness and may have concealed more nuanced attitudinal differences.

Another potential limitation is social desirability bias. As the survey was administered during a mandatory professional seminar, participants may have provided responses perceived as professionally appropriate, particularly regarding patient responsibility and enjoyment of working with patients with eating disorders. However, the anonymous nature of the survey may have reduced this risk.

Other Specified Feeding or Eating Disorder (OSFED) [4] was not included in the current study to avoid uncertainties related to its definition and physicians’ understanding of its diagnostic status. Finally, multiple statistical tests were conducted, which increases the risk of type I errors.

### Implications for practice and research

The findings suggest several implications for primary care practice. Recurrent continuing professional education on eating disorders, clear referral pathways and regular opportunities for consultation with specialist eating-disorder services may improve physicians’ confidence and preparedness in managing these conditions. Particular attention should be directed toward BED, for which physicians reported less clarity regarding their role in the care pathway despite primary care being responsible for managing many of the associated somatic complications. From a healthcare system’s perspective, the findings support continued efforts to strengthen eating-disorder competence within primary care.

Future research would benefit from the development of psychometrically robust instruments for assessing attitudes toward eating disorders. Qualitative or mixed-methods studies may also provide a deeper understanding of physicians’ perceptions of patients with eating disorders. In addition, the findings should be replicated in larger and more geographically diverse samples. Given the limitations of the present study, the findings should be interpreted with appropriate caution until confirmed by future research.

## Conclusions

Primary care physicians reported similar attitudes across eating-disorder diagnoses, although enjoyment of working with this patient group was generally low. Education was consistently associated with greater comfort in management and more positive attitudes, whereas clinical experience was primarily associated with comfort in management. These findings suggest that educational initiatives may play an important role in improving physicians’ preparedness and attitudes toward eating disorders in primary care.

## Appendix 1

### Survey

#### Demographics

Gender:

- Female
- Male
- Other
- Prefer not to answer

Age:

Current level of competence:

- Locum physician prior to internship/foundation training
- Internship/foundation training
- Locum physician after internship/foundation training
- Resident physician in general practice/family medicine
- Resident physician in another specialty
- Specialist in general practice/family medicine
- Specialist in another specialty
- Specialist in two or more specialties

I am currently working at:

- Public primary care center
- Private primary care center

During the past 5 years, I have worked in:

- Maternity care clinic
- Child health care center
- School health services
- Youth health clinic
- None of the above

Number of hours of continuing education regarding eating disorders during the past 5 years:

- 0 hours
- 1–5 hours
- 6–10 hours
- More than 10 hours
- Do not know

How many patients with an eating disorder have you assessed, treated or referred during the past 5 years?

- 0 patients
- 1–5 patients
- 6–10 patients
- More than 10 patients
- Do not know

#### Attitude-questions

Please indicate the extent to which you agree with the following statements regarding individuals with ANOREXIA NERVOSA. Response options: Strongly agree, Agree, Neither agree nor disagree, Disagree, Strongly disagree

- I feel comfortable managing these patients.
- Compared with other patient groups, I enjoy working with these patients.
- Anorexia nervosa is primarily a psychological condition.
- Symptoms of anorexia nervosa are common in the general population and usually resolve over time without specific treatment.
- Anorexia nervosa is often a severe and enduring illness.
- Patients with anorexia nervosa are responsible for their own condition.
- Anorexia nervosa has a major impact on the patient’s quality of life.
- Anorexia nervosa causes difficulties for the patient’s family and friends.
- Effective treatments exist for children and adolescents with anorexia nervosa, but not for adults.
- Patients can do a great deal themselves to control the symptoms of anorexia nervosa.
- I believe that I play an important role in the care of these patients.
- I receive adequate support from colleagues in the management of these patients.
- I have a clear understanding of my role within the care pathway for these patients.

Please indicate the extent to which you agree with the following statements regarding individuals with BULIMIA NERVOSA. Response options: Strongly agree, Agree, Neither agree nor disagree, Disagree, Strongly disagree

- I feel comfortable managing these patients.
- Compared with other patient groups, I enjoy working with these patients.
- Bulimia nervosa is primarily a psychological condition.
- Symptoms of bulimia nervosa are common in the general population and usually resolve over time without specific treatment.
- Bulimia nervosa is often a severe and enduring illness.
- Patients with bulimia nervosa are responsible for their own condition.
- Bulimia nervosa has a major impact on the patient’s quality of life.
- Bulimia nervosa causes difficulties for the patient’s family and friends.
- Treatment is highly effective for bulimia nervosa.
- Patients can do a great deal themselves to control the symptoms of bulimia nervosa.
- I believe that I play an important role in the care of these patients.
- I receive adequate support from colleagues in the management of these patients.
- I have a clear understanding of my role within the care pathway for these patients.

Please indicate the extent to which you agree with the following statements regarding individuals with BINGE EATING DISORDER. Response options: Strongly agree, Agree, Neither agree nor disagree, Disagree, Strongly disagree

- I feel comfortable managing these patients.
- Compared with other patient groups, I enjoy working with these patients.
- Binge eating disorder is primarily a psychological condition.
- Symptoms of binge eating disorder are common in the general population and usually resolve over time without specific treatment.
- Binge eating disorder is often a severe and enduring illness.
- Patients with binge eating disorder are responsible for their own condition.
- Binge eating disorder has a major impact on the patient’s quality of life.
- Binge eating disorder causes difficulties for the patient’s family and friends.
- Treatment is highly effective for binge eating disorder.
- Patients can do a great deal themselves to control the symptoms of the disorder.
- I believe that I play an important role in the care of these patients.
- I receive adequate support from colleagues in the management of these patients.
- I have a clear understanding of my role within the care pathway for these patients.

Please indicate the extent to which you agree with the following statements regarding individuals with AVOIDANT/RESTRICTIVE FOOD INTAKE DISORDER (ARFID). Response options: Strongly agree, Agree, Neither agree nor disagree, Disagree, Strongly disagree

Screening question (gatekeeping question): I am familiar with the diagnosis Avoidant/Restrictive Food Intake Disorder (ARFID) and feel comfortable expressing my views regarding the disorder and individuals with the disorder.

- Yes
- No

If no, you will proceed to the next page.

- I feel comfortable managing these patients.
- Compared with other patient groups, I enjoy working with these patients.
- ARFID is primarily a psychological condition.
- Symptoms of ARFID are relatively common in the general population and usually resolve over time without specific treatment.
- ARFID is often a severe and enduring illness.
- Patients with ARFID are responsible for their own condition.
- ARFID has a major impact on the patient’s quality of life.
- ARFID causes difficulties for the patient’s family and friends.
- Treatment is highly effective for ARFID.
- Patients can do a great deal themselves to control the symptoms of ARFID.
- I believe that I play an important role in the care of these patients.
- I receive adequate support from colleagues in the management of these patients.
- I have a clear understanding of my role within the care pathway for these patients.
